# Exploration of Providers’ Perceptions and Attitudes Toward Phage Therapy and Intentions for Future Adoption as an Alternative to Traditional Antibiotics in the US—A Cross-Sectional Study

**DOI:** 10.3390/ijerph22071139

**Published:** 2025-07-18

**Authors:** Subi Gandhi, Dustin Edwards, Keith Emmert, Bonnie Large

**Affiliations:** 1 Department of Medical Lab Sciences, Public Health, and Nutrition Science, Tarleton State University, 1333 West Washington, Stephenville, TX 76402, USA; 2 Department of Biological Sciences, Tarleton State University, 1333 West Washington, Stephenville, TX 76402, USA; dcedwards@tarleton.edu; 3 Department of Mathematics, Tarleton State University, 1333 West Washington, Stephenville, TX 76402, USA; emmert@tarleton.edu

**Keywords:** phage therapy, antibiotic resistance, antimicrobial resistance, therapeutic alternatives, physician attitudes

## Abstract

Antibiotic resistance presents a global threat, making the swift development of alternative treatments essential. Phage therapy, which employs bacterial viruses that specifically target bacteria, shows promise. Although this method has been utilized for over a century, primarily in Eastern Europe, its use in the US remains limited. This study aimed to assess the awareness and willingness of US healthcare providers to adopt phage therapy in response to the growing issue of antibiotic resistance. A survey of 196 healthcare providers, primarily MDs and DOs, found that while 99% were aware of antimicrobial resistance, only 49% were knowledgeable about phage therapy as a treatment for resistant bacterial infections. Nonetheless, 56% were open to considering phage therapy, and this willingness was associated with prior knowledge, concerns about antibiotic resistance, previous training, and confidence in recommending it (*p* < 0.05). Our study of U.S. healthcare providers revealed key findings about their views on phage therapy as a potential alternative for treating bacterial infections. Credible information is essential to promoting phage therapy use among U.S. providers via educational initiatives, clinical guidance, and research dissemination to promote phage therapy use among U.S. providers. Evidence-based education and clinical guidance help providers make sound decisions on the appropriate and safe use of phage therapy.

## 1. Introduction

Bacterial antibiotic resistance (ABR) poses a significant and growing global health challenge [1,2,3,4]. This resistance occurs when bacteria develop the ability to withstand antibiotics intended to eliminate them [5,6]. Since the advent of antibiotics in the 20th century, they have become a vital cornerstone of modern medicine, saving millions of lives from what were once fatal infections [4,7]. However, the overuse and misuse of antibiotics, coupled with the natural capacity of bacteria to develop resistance through genetic modifications, are critical drivers in the rise of antibiotic-resistant strains [4,8]. In 2019 alone, it was estimated that nearly 5 million global deaths were associated with ABR, while 1.27 million deaths were attributable to ABR [1]. The highest number of ABR-associated deaths across all ages was observed in sub-Saharan Africa, while the lowest was in Australasia, when predictive statistical modeling was employed to estimate the global disease burden in different regions [1]. Among pathogens, the top six main contributors globally were *Escherichia coli*, *Staphylococcus aureus*, *Klebsiella pneumoniae*, *Streptococcus pneumoniae*, *Acinetobacter baumannii*, and *Pseudomonas aeruginosa* [1].

Antimicrobial resistance (AMR) is a major public health issue in the United States [2]. Antimicrobials encompass a wide range of drugs used to treat infections caused by various pathogens, including bacteria and fungi. Conversely, antibiotics are a specific type of drug aimed at treating bacterial infections [9]. The Centers for Disease Control and Prevention (CDC) has identified numerous drug-resistant bacteria as urgent, serious, and concerning threats [5]. Nonetheless, the current pipelines for antibiotics are insufficient to combat the escalating threat of AMR [10]. In 2013, around 2.6 million Americans were infected annually by AMR pathogens, resulting in nearly 44,000 associated deaths [11]. Healthcare Associated Infections (HAI) caused by one of the ESKAPEE pathogens (*Enterococcus faecium*, *Staphylococcus aureus*, *Klebsiella pneumoniae*, *Acinetobacter baumannii*, *Pseudomonas aeruginosa*, *Enterobacter* spp., and *Escherichia coli*) often harbor AMR [12]. The already critical situation is worsened by the slow pace of new antibiotic development and dwindling interest in this area [13]. Antibiotic usage for bacterial coinfections and secondary bacterial infections has increased due to COVID-19 [14]. Therefore, AMR is expected to remain a major public health challenge in the foreseeable future, highlighting the need for innovative and effective strategies to tackle the issue [1,4,13].

Phage therapy has been explored as an alternative to conventional antibiotics [4,15,16]. Bacteriophages, commonly referred to as phages, are natural viruses that specifically target bacteria and can serve as a medical treatment for bacterial infections [4,17,18]. Phages possess several unique traits that distinguish them from antibiotics. They are noted for their greater availability, variability, specificity, low inherent toxicity, effectiveness against antibiotic-resistant bacteria, and minimal environmental impact [4,16]. These exceptional traits render them effective alternatives to antibiotics for therapeutic use. However, phage therapy faces several challenges, including the potential for bacteria to develop resistance to phages, narrow host ranges that limit its applicability to diverse bacterial strains, difficulties in optimizing dosing and delivery, the potential for immune responses such as anti-phage antibody production, and complex regulatory frameworks [19,20,21].

Phages were first identified over a century ago by Felix d’Herrelle and Frederick Twort, and since then, their potential as therapeutic agents has oscillated in popularity [16,22,23]. Currently, phage therapy is not considered the standard treatment in the U.S. or most other nations, primarily due to the scarcity of centers, physicians, and researchers specializing in this field [24,25]. However, recent advances in genetic engineering and phage biology, alongside successful compassionate use cases addressing antibiotic-resistant infections, have rekindled interest in phage therapy [24,26]. Furthermore, the National Institutes of Health has allocated USD 2.5 million to 12 institutes globally to investigate phage therapy, with several clinical trials now in progress to treat various infections [27].

In Eastern Europe, particularly in Georgia, Poland, and Russia, a strong historical and scientific foundation exists for utilizing bacteriophages as a therapeutic agent, despite their decline in popularity in the West [28]. Founded in 1923, the Eliava Institute of Bacteriophages, Microbiology, and Virology in Tbilisi, Georgia, has served as a pivotal center for phage research and production on a global scale [29]. Although there have been challenges, including inconsistent production methods and quality control issues, ongoing experience with phage therapy in Eastern Europe has led to significant scientific insights regarding its potential applications and limitations for treating various antibiotic-resistant infections [30,31]. Given its growing acceptance, phage formulations are now readily available at pharmacies in these countries. They are used to manage conditions such as wound infections, septicemia, and even antibiotic-resistant hospital-acquired infections [19].

Although phage therapy offers a promising approach to combating antibiotic resistance, its clinical uptake remains limited, particularly in the United States, primarily due to knowledge and familiarity gaps among providers [29,30,31]. Several leading U.S. institutions [20,26,32,33,34,35,36] are advancing clinical bacteriophage therapy, including the Center for Innovative Phage Applications and Therapeutics (IPATH) at the University of California San Diego; Tailored Antibacterials & Innovative Laboratories for Φ Research (TAILΦR) at Baylor College of Medicine; the Center for Phage Technology (CPT) at Texas A&M University; the Center for Phage Biology & Therapy at Yale University; the Phage Therapy Program within Mayo Clinic’s Center for Individualized Medicine; the Pittsburgh Phage Program (P3) at the University of Pittsburgh/UPMC; the Department of Defense Bacteriophage Therapeutics Program, based at the Naval Medical Research Center and Walter Reed Army Institute of Research; and the Phage & CRISPR Immunology Group at Johns Hopkins University School of Medicine. Many healthcare professionals remain unaware of phage therapy or only possess a rudimentary understanding of its clinical applications and effectiveness [37]. Despite having a long history, phage therapy has not been extensively incorporated into Western medical education or practice, leading to reluctance and underuse in clinical environments [38,39].

Healthcare providers are strategically positioned to tackle the escalating threat of antimicrobial resistance. Since physicians and other providers, such as physician assistants and nurse practitioners, are the main prescribers of antimicrobials, they have a direct impact on treatment decisions and patient care [40,41,42]. Their comprehension of antibiotic resistance and new alternatives, such as phage therapy, is essential not only for informing individual treatment decisions but also for influencing broader antimicrobial stewardship practices. By enhancing their knowledge and involvement with phage therapy, healthcare providers can promote its responsible integration, uphold evidence-based clinical advancements, and contribute significantly to reducing dependence on conventional antibiotics.

The world currently faces a significant antibiotic shortage driven by insufficient research and development, rising resistance, and an urgent need for equitable access to solutions addressing this issue [3,4]. Additionally, healthcare providers’ understanding, in conjunction with specific policies and regulations, plays a crucial role in the uptake of scientific innovations in clinical practice. Our study aimed to evaluate healthcare providers’ awareness of phage therapy and their readiness to incorporate it into their practices, should it become a viable treatment option in the near future in the United States. To accomplish this, we conducted a nationwide cross-sectional survey targeting medical professionals across all 50 states.

Several key research questions guided the study:

1. What is the present understanding and level of concern among healthcare providers regarding antibiotic resistance?
2. If phage therapy were to become available in the United States, how receptive would providers be to its adoption?
3. Does a provider’s likelihood of considering phage therapy correlate with specific demographic characteristics, knowledge levels, or intention-related factors?

## 2. Materials and Methods

### 2.1. Survey Design, Instrument, Inclusion Criteria, and Ethics

A cross-sectional survey was created to gather data from study participants (providers practicing medicine in various US states). A twenty-item self-administered (see Appendix A) survey was launched via Qualtrics (Qualtrics, Provo, UT) in summer 2021. The survey was designed to be completed in under ten minutes and included a mix of multiple-choice questions, Likert-type scales, and open-ended responses. The survey was divided into three parts, as explained below:

1. Part I: Demographic questions included variables such as age, gender, race, ethnicity, employment status, political affiliation, and marital status.
2. Part II: Profession-related questions inquired about the zip code of respondents’ current practice, the professional degree obtained (e.g., M.D., D.O.), the location of practice, and the year of licensure attainment.
3. Part III: Questions evaluated knowledge of phage therapy and the respondents’ willingness to adopt phage therapy in the future.

### 2.2. Statistical Analysis

Data obtained from Qualtrics surveys were systematically consolidated utilizing the R statistical software (version 4.1.2) to facilitate further in-depth analyses. In this process, various descriptive statistics were calculated, including measures such as the mean, median, frequencies, and percentages for the relevant variables of interest. Additionally, the Chi-square Test of Independence was employed to investigate the relationships of interest, applying an alpha significance level of 0.05 to determine statistical significance in the findings.

### 2.3. Human Subjects Protection

This research received ethical approval from the Institutional Review Board (IRB) at the university where it was conducted, confirming adherence to established ethical guidelines. The research was deemed “exempt” by the IRB. Prior to participating in the Qualtrics survey, all participants were thoroughly informed and provided with comprehensive informed consent documents that outlined the purpose of the study, their role, and their rights. Additionally, participants were given the option to opt out of the study at any point during the survey, ensuring that their participation was completely voluntary. To protect the confidentiality of the participants, all responses were recorded anonymously.

## 3. Results

In total, 225 healthcare providers took part in the survey. The majority held an M.D. degree (71.6%), followed by D.O. degrees (25.8%). Additionally, licensed nurse practitioners made up 1.3%, along with other provider types, also accounted for 1.3%. Table 1 illustrates the demographic variables of the study participants.

**Figure 1:**
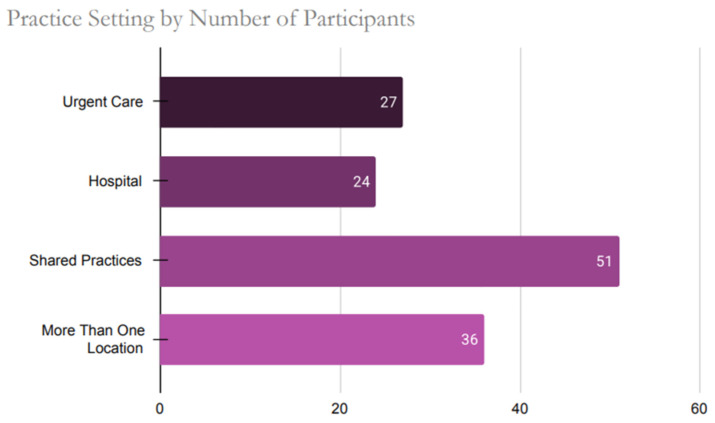
Practice Setting Distribution Among U.S. Healthcare Providers Participating in the Survey.

**Figure 2:**
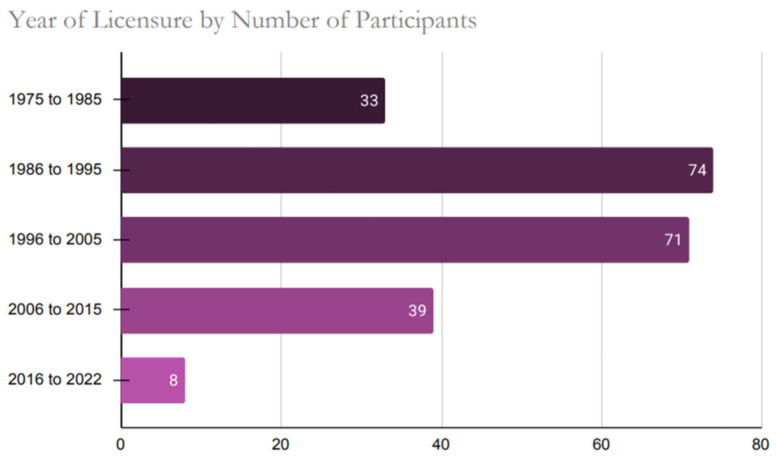
Number of Medical Practitioners Licensed in the U.S. by Decade of Initial Licensure (1975–2022).

Healthcare providers practicing in forty-six out of the fifty states participated in the study. The top three states where the participants’ responses were higher compared to other states were Florida (n = 22), California (n = 18), and Pennsylvania (n = 16). The four non-represented states in the sample were Idaho, Montana, North Dakota, and Vermont. When participants were assessed by zip codes, there were six with the highest number of responses as follows: 33,065 (n = 3, Pompano Beach, FL), 60,101 (n = 3, Addison, IL), 17,603 (2, Lancaster, PA), 23,228 (n = 2, Richmond, VA), 55,905 (2, Rochester, MN) and 76,904 (n = 2, San Angelo, TX). Figure 1 below illustrates the diverse range of practice facilities among healthcare providers across the US, while Figure 2 presents the distribution of licensure attainment by decade.

When inquired about their familiarity with “phage therapy” as a treatment option for resistant bacterial infections in either animals or humans, 49% (n = 110) of the providers replied “yes.” In contrast, 32% (n = 72) answered “no,” while 19% (n = 43) expressed uncertainty. Among those aware of phage therapy, 66 learned about it through conferences, 39 through their clinical practices, and 25 from professional schools. Additionally, providers noted the following as “other” sources: news articles, casual readings, medical journals or newsletters, internet resources, and medical news websites.

**Figure 3:**
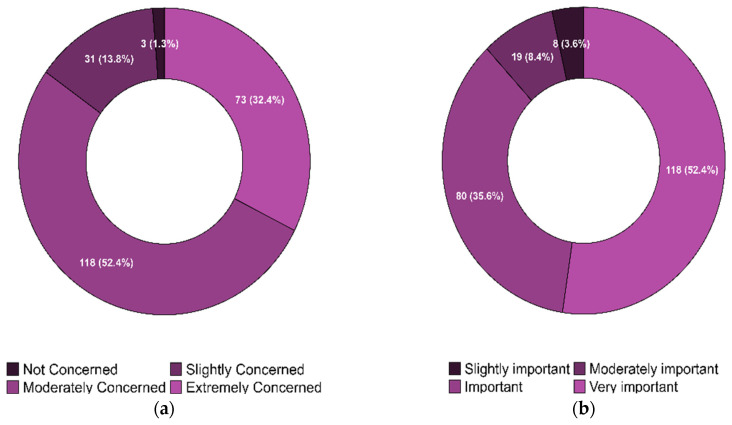
(**a**). Physicians’ Concern about the Current State of Antibiotic Resistance in the United States. (**b**). Physicians’ Perceptions of the Importance of Finding an Alternative Therapy for Antibiotic Resistance in the United States.

**Figure 4:**
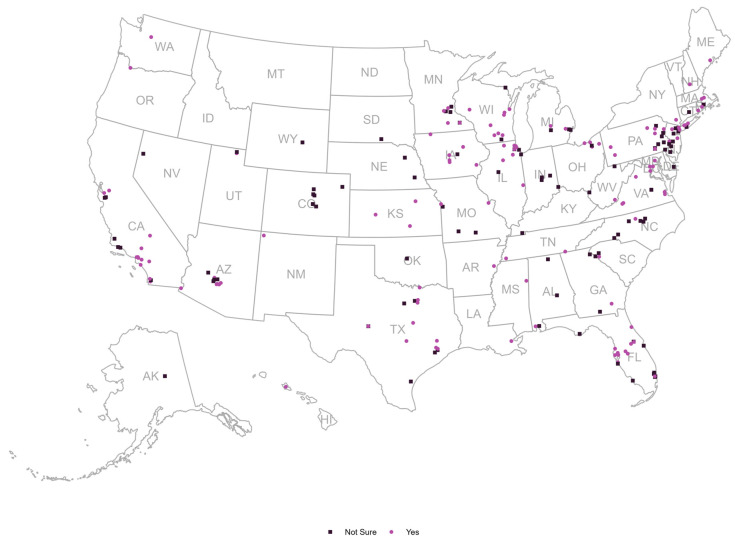
Geographic Distribution of Healthcare Providers’ Willingness to Use Bacteriophage Therapy in the U.S. (n = 225) [Note: Each dot corresponds to a survey respondent representing their respective state].

Nearly all (99%) providers had previously been aware of antibiotic resistance in their practices. The responses regarding the providers’ concerns about the current state of antibiotic resistance in the U.S. and their perceptions of the importance of developing alternative therapies for antibiotic resistance are illustrated in Figure 3a and Figure 3b, respectively. Figure 4 shows the distribution of providers based on their willingness to adopt phage therapy in the future, by zip code. At the time of the survey, 125 physicians were willing to adopt phage therapy, and 100 were undecided.

**Figure 5:**
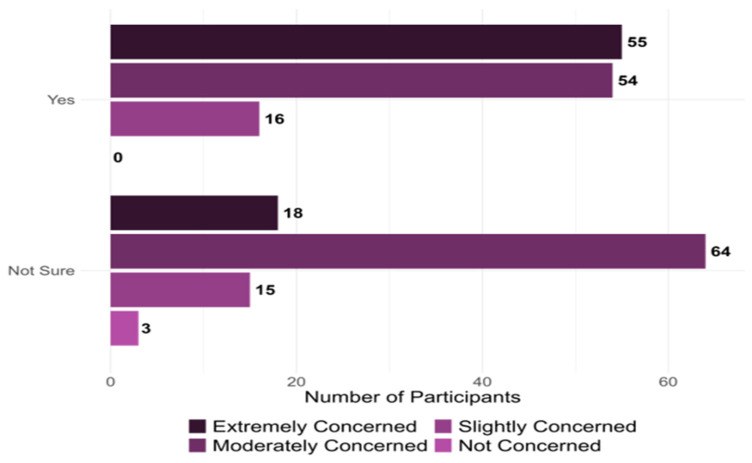
Provider Willingness to Adopt Phage Therapy and Perceived Concerns Regarding Antibiotic Resistance (n = 225).

Figure 5 illustrates the likelihood of integrating phage therapy into their practice, influenced by their concern about the current state of antibiotic resistance, measured on a Likert-type scale (1 = not concerned to 4 = extremely concerned). For those who are willing to adopt phage therapy, 55 (24.4%) are extremely concerned, 54 (24%) moderately concerned, 16 (7.1%) slightly concerned, 0 (0%) are not concerned. The association between critical variables and physicians’ future adoption of phage therapy is detailed in Table 2.

**Figure A1:**
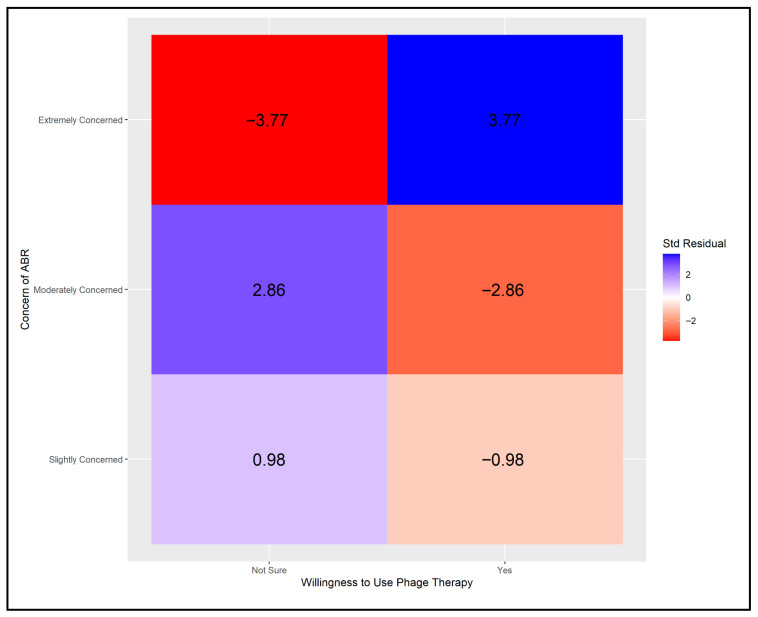
Adjusted Pearson residuals for physicians’ willingness to use phage therapy by level of concern about ABR [Critical value ± 2.39 (Bonferroni-adjusted α = 0.05/6)].

**Figure A2:**
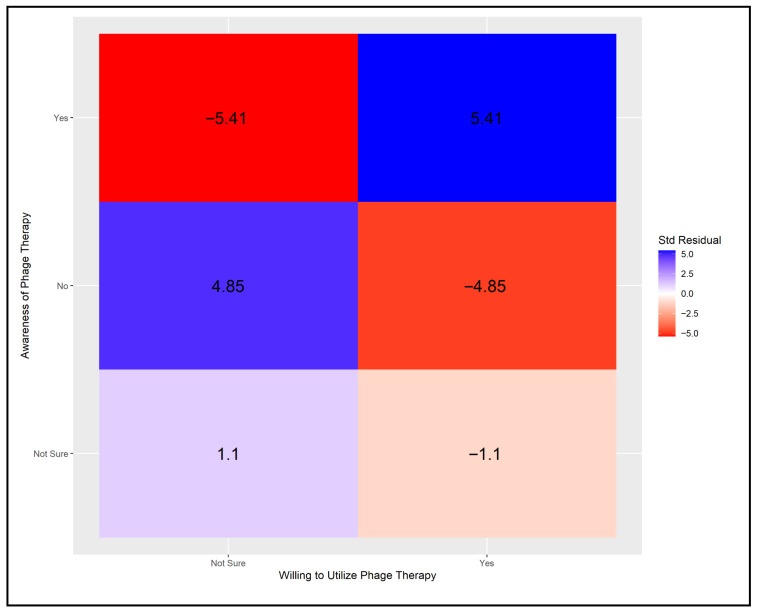
Adjusted Pearson residuals for physicians’ willingness to use phage therapy by level of awareness [Critical value ± 2.39 (Bonferroni-adjusted α = 0.05/6)].

**Figure A3:**
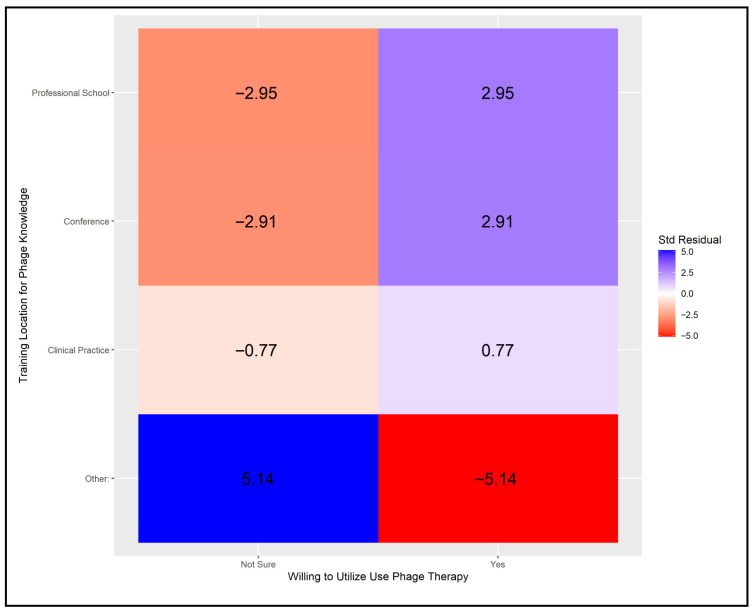
Adjusted Pearson residuals for physicians’ training location in relation to their knowledge and willingness to use phage therapy [Critical value ± 2.5 (Bonferroni-adjusted α = 0.05/8)].

**Figure A4:**
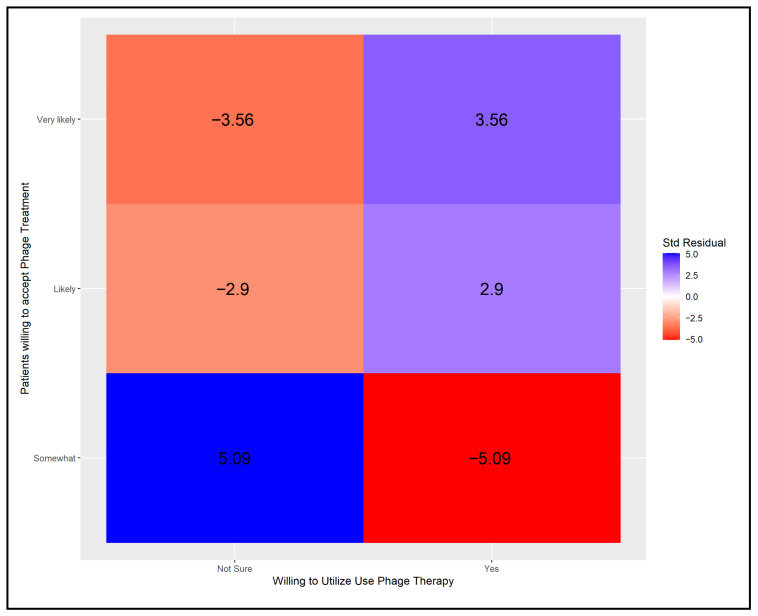
Adjusted Pearson residuals for physicians’ willingness to use phage therapy and their perceptions of patients’ willingness to accept it [Critical value ± 2.39 (Bonferroni-adjusted α = 0.05/6)].

When the $\chi^{2}$ independence tests indicated a rejection of the null hypothesis, we examined the adjusted Pearson residuals. These residuals, based on an alpha level of 0.05 and adjusted for the number of cells in each contingency table, helped identify significant deviations from expected cell counts. Further details and illustrative examples can be found in Appendix B (Figure A1, Figure A2, Figure A3 and Figure A4).

Physicians’ likelihood to employ phage therapy, in conjunction with their level of worry ABR, reveals significant findings. Notably, instances where ABR concern is moderate or extreme display statistical relevance. Extreme ABR concern shows the most substantial deviations from expected counts. The highest positive deviation from the expected count is observed when physicians exhibit a strong willingness to utilize phage therapy alongside extreme ABR concern. Furthermore, moderate ABR concern paired with physician uncertainty about using phage therapy yields the next highest positive deviation. These findings are visually represented in Figure A1.

Statistical analysis revealed significant variations between physicians’ willingness to use phage therapy and their level of awareness. Notably, a greater number of physicians than expected were both willing to utilize phage therapy and possessed positive awareness of it. Conversely, the number of physicians willing to use phage therapy despite negative awareness was lower than anticipated. This relationship is illustrated in Figure A2.

Physicians with formal training in phage therapy, such as those that attended conferences or professional schools, demonstrated a statistically higher willingness to use it. In contrast, those who learned about phage therapy through means other than professional education and clinical experience generally showed hesitation towards its application. This is visually represented in Figure A3.

In physicians open to phage therapy, higher counts were observed than expected in the “likely” or “very likely” categories for patients deemed receptive to such treatment. Conversely, when physician uncertainty regarding phage therapy existed, a statistically significant increase in physicians predicting their patients would be “somewhat likely” to accept it was noted. These correlations are depicted in Figure A4.

## 4. Discussion

The rise of antibiotic resistance in bacteria has become a global concern, and the impact of these infections on morbidity and mortality is projected to grow in the foreseeable future unless a promising alternative emerges. The renewed interest in phage therapy as an alternative signifies a significant revival in treating drug-resistant infections [18,28,43,44]. It has gained considerable attention in recent years as a potential substitute for traditional antibiotics. Despite this growing interest, the Food and Drug Administration (FDA) has not yet approved phage therapy in the United States. This is primarily due to the need for comprehensive clinical data to establish the treatment’s safety and efficacy. Nevertheless, progress is being made in the field, as the first clinical trial investigating the intravenous administration of phage therapy has received approval [43]. This trial represents a crucial step forward in evaluating phage therapy’s therapeutic potential for patients with serious bacterial infections, indicating that regulatory pathways may be opening for this innovative treatment option.

Given the increasing international medical interest in phage therapy, it is a suitable time to investigate the diverse aspects of phage therapy within the United States [43]. This timely and relevant investigation aligns with the international trend of heightened attention toward this therapeutic approach. In addressing antimicrobial resistance infections, the role of healthcare providers as primary facilitators of communication and proponents of antimicrobial therapies is increasingly critical. Their comprehensive understanding of antimicrobial stewardship and capacity to effectively persuade patients to embrace appropriate therapeutic interventions are pivotal, particularly at this crucial juncture. This underscores the necessity for enhancing providers’ expertise in antimicrobial knowledge and their confidence in guiding patients toward adopting these therapies to treat ABR infections [38,39].

Although research on this topic has been carried out in other countries, no US-based study has yet captured providers’ perspectives on adopting or future practices of phage therapy. For instance, a recent survey of clinicians in the UK revealed that approximately 59% had heard of phage therapy, and over 70% would consider using it in suitable cases. [18]. In Australia, 97% of infectious disease specialists expressed willingness to use phage therapy if high-quality, regulated preparations were accessible. However, many pointed out practical barriers, such as the need for timely access to phages (mentioned by 72% of respondents) and logistical challenges in procurement [45]. Surveys in Poland have revealed relatively modest baseline knowledge of phage therapy among healthcare providers—only about one-third of physicians and dentists expressed a readiness to deepen their phage knowledge—yet an overwhelming majority of respondents (84.4%) said they would undergo phage treatment if needed, even if it meant paying out of pocket [46]. Similarly, Korean infectious disease specialists showed limited awareness of phage therapy (only a few felt well-informed), but most were still eager to participate in phage therapy clinical trials, with concerns centered on safety, efficacy, and logistical challenges [47]. In the United States, formal survey data are limited, but reports from clinical centers like UC San Diego’s IPATH indicate a significant awareness gap—patients reportedly request phage therapy more often than physicians provide it [48]. Overall, across different countries, general awareness of phage therapy among providers remains variable and often limited, but the attitude toward phage therapy is broadly positive; most physicians are open to or enthusiastic about phage use for ABR infections. This study represents the first comprehensive national assessment of healthcare providers’ knowledge and attitudes toward phage therapy in the United States. By examining provider perspectives on this emerging treatment, we offer critical insights into the potential of phage therapy as an alternative strategy for combating ABR infections.

Our survey of U.S. healthcare providers revealed meaningful insights into their perceptions of phage therapy as an emerging alternative for treating bacterial infections. Participants represented diverse professional backgrounds, with a majority holding M.D. degrees and working in private practice. Most respondents acknowledged the growing challenge of antibiotic resistance and reported some familiarity with phage therapy, often through professional conferences or clinical experiences. Encouragingly, a notable proportion expressed interest in incorporating phage therapy into their future practice. However, many providers remained uncertain, likely due to limited exposure, concerns about clinical efficacy, ethical concerns, and the lack of standardized regulatory guidance [18,49]. Importantly, nearly one-third of providers had not previously encountered phage therapy as a treatment option, pointing to significant gaps in awareness and training.

These findings are consistent with previous studies that have identified a lack of knowledge about bacteriophage therapy among healthcare providers as a key barrier to its wider acceptance and use [31,50,51]. Our data corroborates this, with only about half of the respondents reporting prior knowledge of phage therapy. When comparing global perspectives, our findings indicate a lower level of readiness among U.S. providers to adopt phage therapy than their international counterparts. For example, approximately 70% of clinicians in Canada and the UK have reported their willingness to use phage therapy in appropriate cases [18,52]. At the same time, an Australian survey found that 97% would consider its use, provided it met established safety and purity standards [45]. Notably, higher acceptance rates in these studies may reflect greater exposure or institutional familiarity with phage therapy, particularly in the UK, where respondents had prior clinical experience with it, potentially inflating perceived acceptance [18].

The current understanding of phage therapy within the U.S. healthcare system is varied, yet amid growing concerns about antibiotic resistance, there is a noticeable shift in provider attitudes. Many healthcare professionals are increasingly receptive to alternatives, viewing phage therapy as a potential complement, or even a replacement, in specific clinical scenarios. This rising awareness is likely influenced by exposure to professional development opportunities and firsthand clinical experiences. However, significant hesitancy remains, largely due to limited access to robust clinical data, unclear regulatory pathways, and uncertainty about practical implementation.

To reduce ambiguity and support the appropriate adoption of phage therapy, it is essential that American healthcare providers have access to reliable, evidence-based information. Educational initiatives, both online and in-person, along with clear clinical guidelines and hands-on training, are critical. Enhancing provider education, expanding the dissemination of research, and integrating phage therapy into ongoing medical education will not only improve individual patient outcomes but also contribute meaningfully to the broader public health effort to combat antibiotic resistance. Transitioning phage therapy from an experimental concept to a viable clinical tool hinges on making such resources widely accessible and actionable.

Our study presents findings that may inform future research and practice, while acknowledging several key limitations. While our sample size is considerable, it may not fully capture the diversity of healthcare providers throughout the US. The number of physicians involved in each state was limited, which likely restricted the representation of the average opinions and beliefs of healthcare providers both within individual states and across all states. Furthermore, our research relies on self-reported data, which are vulnerable to biases related to recall and social desirability [53]. Another limitation is that, although we identified a correlation between previous awareness of phage therapy and future willingness to adopt it, we cannot definitively establish causality due to the study’s cross-sectional design.

Social and cultural factors, such as political ideology, significantly influence health decisions [54,55]. An example of this concept is readily illustrated by the vaccine uptake rates at the population level [54,55]. A study on vaccine confidence among primary care physicians during the COVID-19 pandemic suggested that conservative physicians might be less likely to present information about the benefits of vaccination, especially in areas where vaccine hesitancy and conservative views are prevalent [56]. The same study also noted a broader issue related to vaccine hesitancy among physicians: limited training on vaccines and vaccinology in medical school, which may lead to less well-formed opinions on vaccines. This shortfall in training, combined with the misinformation-laden environment surrounding COVID-19 and vaccines, suggests that some physicians may rely more on news and misinformation than on scientific evidence. Literature has demonstrated that medical conservatism among physicians can limit medical advancements [57,58]. In our study, we were unable to demonstrate the association between political affiliation and the intentionality of adopting phage therapy. However, this finding contributes to the ongoing discourse on the complex relationship between political beliefs, healthcare decision-making, and the acceptance of new treatment modalities, such as phage therapy, and should be supported by future robust studies.

The future understanding of phage therapy acceptance in the US involves several crucial elements, including public perception, access to education, and media portrayal. A prior study in the UK revealed limited awareness and inadequate understanding of phage therapy [18]. However, a related framing experiment demonstrated that even slight exposure to information on antibiotic resistance and alternative treatments can significantly enhance public acceptance of phage therapy. Participants also expressed a keen interest in the need for increased public education on the subject [18]. The SEA-PHAGES program, supported by the Howard Hughes Medical Institute, plays a vital role in advancing the acceptance of phage therapy in the US by improving educational access [49,50,51,52,53,54]. By involving over 5500 undergraduate students each year in phage discovery and genomics, the program nurtures a deeper understanding and interest in phage therapy among tomorrow’s scientists and healthcare providers. This hands-on experience equips students with knowledge about the potential of phage therapy, preparing them to be informed advocates in their future roles. Such extensive educational efforts build a knowledge base that can enhance the acceptance and implementation of phage therapy into the broader community. Additionally, raising public awareness and understanding of phage therapy could benefit from media coverage and the sharing of success stories. For example, scientists featured on popular platforms like CNN help clarify phage therapy and showcase its potential as a promising alternative to conventional antibiotics, particularly in addressing antibiotic-resistant infections [55]. Future surveys could assess the current public awareness of phage therapy in the US, focusing on the impact of media coverage, common misconceptions, and knowledge gaps to inform effective communication strategies. Furthermore, exploring how educational programs impact students’ attitudes towards phage therapy would yield insights into the influence of education on the perspectives of future healthcare professionals.

## 5. Conclusions

Our research shows a positive outlook among healthcare providers toward phage therapy despite its limited use. The gap between interest and application highlights the need for educational initiatives to increase awareness and understanding of phage therapy as a viable alternative for treating bacterial infections. Addressing potential barriers to widespread use should be a priority for the healthcare community. Future research should focus on comprehensively understanding these barriers. Further studies are also required to confirm the correlation between years of licensure attainment and the willingness to adopt phage therapy. As antibiotic resistance worsens globally, it is critical to explore and accept alternative treatments. The study evaluated providers’ knowledge of phage therapy, their exposure to relevant information during professional development, and their willingness to adopt this treatment in the future. We aimed to determine how demographic and professional characteristics shape these perceptions. The data gathered provide insights into healthcare providers’ understanding of phage therapy and implications for policy and practice decisions in US primary care settings.

## Figures and Tables

**Table 1:** Basic demographic characteristics of the study participants (n = 225).

Characteristics	Mean	Std Deviation
Age (Years)		
Overall	54.29	9.99
Male	55.72	9.91
Female	51.38	9.68
	Frequency (n)	Percentage (%)
Gender		
Male	148	65.8
Female	74	32.9
Others	3	1.3
Race		
White	171	76.0
Black	6	2.7
Asian	36	16.0
Others	12	5.3
Ethnicity		
Hispanic or Latino	4	1.8
Non-Hispanic or Latino	221	98.2
Employment Status		
Employed full-time	206	91.6
Employed part-time	14	6.2
Retired and other	5	2.2
Political Affiliation		
Conservative (very)	11	4.9
Conservative (moderate)	43	19.1
Conservative (light)	28	12.4
Neutral	50	22.2
Liberal (light)	23	10.2
Liberal (moderate)	34	15.1
Liberal (very)	10	4.4
Prefer not to answer	26	11.6
Marital Status		
Married/Not married, but cohabiting	189	84.0
Married, but not cohabitating	4	1.8
Not married or cohabitating	29	12.9
Other	3	1.3
Professional Degree		
Doctor of Medicine (MD)	161	71.6
Doctor of Osteopathic Medicine (DO)	58	25.8
Others (e.g., DPM, LNP)	6	2.7

**Table 2:** The relationship between specific variables and physicians’ future willingness to adopt phage therapy (n = 216).

	Willing N (%)	Unsure N (%)	𝜒^2^	*p*
Age (years)			0.286	0.59
<50	39 (18.1)	26 (12.0)		
≥50 years	83 (38.4)	68 (31.5)		
Gender			0.398	0.53
Male	84 (38.9)	60 (27.8)		
Female	38 (17.6)	34 (15.7)		
Race			0.681	0.71
White	92 (42.6)	73 (33.8)		
Asian	20 (9.3)	16 (7.4)		
Others	10 (4.6)	5 (2.3)		
Employment Status			0.110	0.74
Employed full-time	113 (52.3)	85 (39.4)		
Employed part-time, retired, other	9 (4.2)	9 (4.2)		
Political Affiliation			4.73	0.19
Conservative	35 (16.2)	40 (18.5)		
Neutral	32 (14.8)	18 (8.3)		
Liberal	41 (19.0)	26 (12.0)		
Prefer not to answer	14 (6.5)	10 (4.6)		
Marital Status			1.035	0.31
Married/Not married, but cohabiting	99 (45.8)	82 (38.0)		
Married, but not cohabitating/Not married or cohabitating	23 (10.6)	12 (5.6)		
Year of Licensure Attainment			1.304	0.25
Before 1999	70 (32.4)	62 (28.7)		
After 2000	52 (24.1)	32 (14.8)		
Location of Practice			0.000	1.00
Rural zip codes	16 (7.4)	12 (5.6)		
Non-rural zip codes	98 (45.4)	75 (34.7)		
Providers’ Concerns about Antibiotic Resistance *			14.211	0.00 *
Slightly Concerned	15 (6.9)	16 (7.4)		
Moderately Concerned	54 (25.0)	60 (27.8)		
Extremely Concerned	53 (24.5)	18 (8.3)		
Providers’ Knowledge of Phage Therapy			31.912	0.00 *
Yes	79 (36.6)	26 (12.0)		
No	23 (10.6)	47 (21.8)		
Not Sure	20 (9.3)	21 (9.7)		
Event/Training Where Providers Learned About Phage Therapy			29.283	0.00 *
Professional School	21 (9.7)	4 (1.9)		
Conference	44 (20.4)	17 (7.9)		
Clinical Practice	23 (10.6)	14 (6.5)		
Other	34 (15.7)	59 (27.3)		
Patients Willing to Accept Phage Therapy If Endorsed By Physicians *			30.04	0.00 *
Very likely	21 (9.7)	2 (0.9)		
Likely	63 (29.2)	30 (13.9)		
Somewhat	38 (17.6)	62 (28.7)		

* [Note: 11 physicians have already adopted phage therapy, 200 have not, and 14 are not sure.; Some categories were omitted due to zero responses].

## Data Availability

The original data presented in the study are openly available in “Bacteriophage Therapy Perceptions and Attitudes Towards Therapy”, Mendeley Data, V1, doi: 10.17632/3gyrjdgk4y.1.

## Appendix A. Questionnaire

I. Demographics

1. What is your age in years?

_____________(years)

2. What is your gender identity

○ Male
○ Female
○ Transgender man
○ Transgender women
○ Gender non-conforming
○ Intersex
○ Other
○ Prefer not to answer

3. Race

○ White/Caucasian
○ Black or African American
○ Middle Eastern or North African
○ Native American, American Indian, or Alaskan Native
○ Asian
○ Native Hawaiian or Pacific Islander
○ Two or more races
○ Other/Unknown
○ Prefer not to answer

4. Ethnicity

○ Hispanic or Latino
○ Non-Hispanic or Latino

5. What is your employment status?

○ Employed full-time
○ Employed part-time
○ Retried

6. What is your political affiliation?

○ Very conservative
○ Moderately conservative
○ Lightly conservative
○ Neutral
○ Lightly liberal
○ Moderately liberal
○ Very liberal
○ Prefer not to answer

7. What is your marital status?

○ Married
○ Marries, but not cohabitating
○ Not married, but cohabiting
○ Not married or cohabitating

II. Profession-related questions

8. What is your zip code of practice?

__________________________________

9. What is your professional degree?

○ Doctor of Medicine (MD)
○ Doctor of Osteopathic Medicine (DO)
○ Podiatrist (DPM)
○ Licensed Nurse Practitioner (LNP)
○ Other_________________

10. What is your primary location of practice? Select those that apply.

○ Hospital
○ Private practice/clinic
○ Urgent care clinic
○ Hospice
○ Ambulatory surgical center
○ Group practice
○ Skilled nursing home
○ Nursing home
○ Home health
○ Other (please explain) ________________________

11. In which year did you achieve a license or licensure to practice medicine (Example—1970, 1985)?

______________
III. Antibiotic Resistance and Phage-related Questions

12. Have you heard of the term “antibiotic resistance” before this survey?

○ Yes
○ No

13. How concerned are you about the current state of antibiotic resistance and its public health burden in the US?

○ Not concerned
○ Slightly concerned
○ Moderately concerned
○ Extremely concerned

14. How important do you feel about finding an alternative therapy option for treating antibiotic-resistant bacteria (ARB) in the US?

○ Very Important
○ Important
○ Moderately Important
○ Slightly Important
○ Not Important

15. Have you heard of “bacteriophage therapy” or “phage therapy” as an alternative method to treat resistant bacterial infections in animals and/or humans?

○ Yes
○ No
○ Not sure

16. Where did you learn about “bacteriophage therapy” or “phage therapy”?

○ Professional school
○ Conference
○ Clinical Practice
○ Other (please explain) ________________________

17. Will your patients accept “bacteriophage therapy” or “phage therapy” if you recommend them as an alternative therapy to treat their resistant bacterial conditions?

○ Very likely
○ Likely
○ Somewhat
○ Not at all

18. I am willing to treat patients with “bacteriophage therapy” or “phage therapy” instead of traditional antibiotics when necessary, in the future.

○ Yes
○ No
○ Not sure

19. I am willing to learn more about “bacteriophage therapy” or “phage therapy” in the near future for consideration as an alternative therapy in my practice.

○ Very likely
○ Likely
○ Somewhat
○ Not at all

20. I am already using “bacteriophage therapy” or “phage therapy” in my clinical practice.

○ Yes
○ No
○ Not sure

Thank you for your participation in this survey!

## Appendix B

### Appendix B.1

For those $\chi^{2}$ tests for independence that are rejected at the α=0.05 level, we used the adjusted Pearson residuals to examine each cell in the table for statistically significant contributions to the rejection of the null hypothesis. The adjusted Pearson residuals were calculated using

$$r_{ij}=\frac{O_{ij}-E_{ij}}{\sqrt{E_{ij}(1-m_{i}\;/\;N)(1-n_{j}\;/\;N)}}$$

where $O_{ij}$ and $E_{ij}$ represent the observed and expected counts in row *i* and column *j*, respectively, $m_{i}$ and $n_{j}$ represent the number of row and column totals, respectively, and *N* represents the overall total. Since $r_{ij}$ follows a standard normal distribution, we can calculate critical values, *CV*, based upon an adjusted level α=0.05 / (number of cells). If $r_{ij}>CV$ or $r_{ij}<-CV$, then that cell is significantly different from the expected count under the null hypothesis of independence. Specifically, if $r_{ij}>0$, it indicates that there are more observed values than expected. Conversely, if $r_{ij}<0$, then there are fewer observed than expected. In Figure A1, Figure A2, Figure A3 and Figure A4, cells that are bluer indicate a positive $r_{ij}$, while cells that are redder indicate a negative $r_{ij}$.

The analysis first examined physicians’ concerns regarding antibiotic-resistant bacteria in relation to their willingness to utilize phage therapy. The critical value for significance was set at ±2.39, based on a Bonferroni-adjusted alpha level of α = 0.05/6. As shown in Figure A1, four cells demonstrated significant deviations from expected frequencies. Statistically significant contributions were identified when the standardized residual $r_{ij}$ exceeded 2.39. This pattern was evident among physicians who reported a willingness to try phage therapy and expressed high concern about antibiotic resistance, as well as those who were uncertain about trying phage therapy but exhibited moderate concern. Conversely, $r_{ij}$ values below −2.39 indicated statistically significant underrepresentation among physicians who were moderately concerned about antibiotic resistance yet willing to try phage therapy, and those who were extremely concerned but uncertain about using phage therapy.

The analysis next considered physicians’ awareness of phage therapy in relation to their willingness to use it. The critical value for significance was set at ±2.39, based on a Bonferroni-adjusted alpha level of α = 0.05/6. As shown in Figure A2, four cells demonstrated statistically significant deviations from expected values. The pattern of results was similar to that observed in Figure A1, although the adjusted Pearson residuals were more pronounced.

Next, we examined the association between physicians’ willingness to use phage therapy and the type and setting of their prior training on the topic. A critical value of ±2.5 was applied, based on a Bonferroni-adjusted alpha of α = 0.05/8. As illustrated in Figure A3, six cells showed statistically significant deviations from expected frequencies. Physicians trained through conferences or professional schools and willing to adopt phage therapy contributed positively to the model, while those uncertain about its use showed significant negative contributions from the same training sources. The most pronounced effects were observed among those who received training through alternative venues, where uncertainty was associated with significant positive contributions and willingness with significant negative contributions.

Finally, we examined the relationship between physicians’ willingness to use phage therapy and their perceptions of patients’ willingness to accept such treatment. Results are presented in Figure A4. All cells showed statistically significant contributions, indicating rejection of the null hypothesis. Positive contributions were observed when physicians were willing to use phage therapy and believed their patients were likely or very likely to accept it. In contrast, a significant negative contribution occurred when physicians were willing but perceived their patients as only somewhat likely to accept treatment. Among physicians who were uncertain about using phage therapy, the pattern of contributions swapped.
